# Landscape of Oncology Clinical Trials in Africa

**DOI:** 10.1200/JGO.19.00189

**Published:** 2020-07-02

**Authors:** Folakemi T. Odedina, Delva Shamley, Ifeoma Okoye, Adaora Ezeani, Ntokozo Ndlovu, Yvonne Dei-Adomakoh, Kimberly Meza, Ruth Agaba, Parisa Fathi, Nissa Askins

**Affiliations:** ^1^ University of Florida, Orlando, FL; ^2^ Prostate Cancer Transatlantic Consortium, Orlando, FL; ^3^ University of Cape Town, Cape Town, South Africa; ^4^ University of Nigeria, Nsukka, Nigeria; ^5^ University of Zimbabwe College of Health Sciences, Harare, Zimbabwe; ^6^ University of Ghana, Accra, Ghana

## Abstract

**PURPOSE:**

The burden of cancer in Africa is of significant concern for several reasons, including that incidence of cancer in Africa continues to rise while Africa is also dealing with communicable diseases. To combat cancer in Africa, oncology clinical trials are needed to develop innovative interventions for cancer prevention, screening, diagnosis, treatment, and survivorship. Unfortunately, there is a paucity of clinical trials in Africa and it is difficult for African clinicians to get information on open oncology clinical trials and impossible for African patients with cancer to access this information. The primary objective of this study was to identify open oncology clinical trials in Africa.

**METHODS:**

This project was part of a large-scale study to develop an African Virtual Platform for Oncology Clinical Trials Registry. The study was a quantitative, web-based, retrospective review of clinical trials registries.

**RESULTS:**

A total of 109 open oncology clinical trials were identified. Most of the trials were in Egypt, South Africa, Algeria, and Kenya. The top cancer types for oncology clinical trials in Africa were breast, cervical, and lung cancers. The top sponsor of oncology clinical trials in Africa was academic institutions, especially institutions in the United States.

**CONCLUSION:**

The paucity of clinical trials in Africa will continue to magnify the global disparities of cancer in the African population. Clinical trials are needed to ensure therapeutic interventions are safe and effective in the African population. In the era of personalized and precision health, it no longer suffices to assume that drugs developed in North America, Europe, or Asia will be effective in the African population.

## INTRODUCTION

### Oncology Clinical Trials in Africa: The Need and Barriers

Globally, more than 60% of new cancer cases diagnosed annually occur in Africa, Asia, and Central and South America. About 70% of the world’s cancer deaths also occur in these continents.^1^ Cancer burden in Africa continues to increase while Africa is also dealing with communicable diseases. With Africa containing the second largest population in the world and 25% of the world population is estimated to live in Africa by 2050, cancer in Africa has been appropriately labeled a runaway train. To combat cancer in Africa, oncology clinical trials are needed to develop innovative interventions for cancer prevention, screening, diagnosis, treatment, and survivorship. Unfortunately, there is insufficient representation of Africans in oncology clinical trials globally, thereby limiting optimal cancer prevention, diagnosis, and treatment decisions for Africans.^2-7^

**CONTEXT**

**Key Objective**
To identify and characterize open oncology clinical trials in Africa.
**Knowledge Generated**
We identified 109 open oncology clinical trials in Africa.
**Relevance**
This study is a step towards making oncology clinical trials accessible to cancer clinicians, patients, and the general public in Africa.

There is a crucial need to develop innovative strategies for prevention and early detection of cancer and to ensure access to low-cost anticancer therapies, which are country and region specific. Unfortunately, clinical trials in Africa is an emerging field. An analysis of the numbers of registered clinical trials in different parts of the world from 2004 to 2013 showed the numbers of registered clinical trials were lowest in Africa, Latin America, and the Caribbean regions.^8^ The challenges of conducting clinical trials in Africa are formidable and multifaceted. Some of the common barriers that have been identified for the limited number of clinical trials in Africa include the lack of dedicated research teams, absence of reliable Internet access, limited staff skilled in research, lack of team commitment, inadequate funding, and low prioritization of research.^9^ The conduct of high-impact research requires adequate capacity and regulatory functions to be in place. Quality assurance in clinical trials is of utmost importance and minimum standards are needed at various levels. This can be a challenge in conditions of limited human, financial, and infrastructural resources.^10^

Another significant barrier is the capability of the clinical research team to meet international Good Clinical Practice (GCP) standards, including institutional review boards (IRBs) and ethical issues. Validity of the informed consent process, an important factor in research, is a reason for concern in some studies conducted in Africa. Readability and understandability of the usually long informed-consent document has been disputed, considering the varying levels of literacy among participants and numerous languages and dialects for the translation of the documents.^11,12^ Specific to oncology clinical trials in Africa is that patients with cancer are a vulnerable population with unique needs.^13^ Therapeutic misconception is common: Patients agree to participate in clinical trials without being aware of nonbeneficence but believing they will obtain better quality treatment through the clinical trials because ancillary care is scarce in limited resource settings such as Africa.^14^

### Study Rationale and Objective

Currently, there is heightened interest in oncology clinical trials in Africa to improve patient care, primarily because of limitations in pathology, surgery, medical oncology, radiation, and palliation leading to worse cancer outcomes on the continent. Local high level of evidence to guide cancer treatment in Africans is much needed but scarce.^15^ In some countries, innovative ways are being applied to stimulate an interest in conducting high-quality research that is able to influence policy and stimulate government commitment to support clinical trials.^16^

For the open oncology clinical trials in Africa, a persistent challenge is increasing patient access to these trials. In the United States, oncology providers and patients can easily get information online, either through ClinicalTrials.Gov or websites of cancer centers, about open clinical trials close by. In comparison with the United States, it is difficult for African clinicians to get information on open oncology clinical trials and impossible for patients with cancer in Africa to access this information. Our long-term goal is to make oncology clinical trials accessible to cancer clinicians, patients, and the general public in Africa. In line with this goal, the primary objective of this study was to identify open oncology clinical trials in Africa. Specifically, the following questions were answered to meet this objective: (1) What are the public and private registries for oncology clinical trials in Africa? (2) Who are the sponsors of oncology clinical trials in Africa? (3) What are the common cancer types for oncology clinical trials in Africa?

## METHODS

This project was part of a large-scale study to develop an African Virtual Platform for Oncology Clinical Trials Registry. The study was a quantitative, web-based, retrospective review of clinical trials registries. All 54 African countries were included in this study.

### Databases Searched

We reviewed online clinical trial registries for all open clinical trials in Africa. A search of private and public funding agencies, including pharmaceutical companies, was carried out between May and August 2019. The online registries included Clinicaltrials.gov (http://clinialtrials.gov), Pan African Clinical Trials Registry (http://www.pactr.org), CenterWatch (http://centerwatch.com), Florida Cancer Specialists and Research Institute (https://flcancer.com/en), Ministry of Health, Burkina Faso (http://www.sante.gov.bf), South African National Clinical Trial Register (www.sanctr.gov.za), World Health Organization International Clinical Trials Registry Platform (http://apps.who.int/trialsearch/Default.aspx), Regulatory Intelligence Sources (https://www.reg-info.com/other/africa), and European Union Clinical Trials Register (https://www.clinicaltrialsregister.eu). In addition, the websites of pharmaceutical companies with oncology products, websites of African Ministries of Health, and websites of African Regulatory agencies were included in the search. Finally, search engines such as Google and Yahoo also were used.

### Search Terms

The search terms for this study were: “name of country,” “Africa,” “oncology,” “cancer,” and “clinical trials.”

### Inclusion and Exclusion Criteria

The African population was the targeted population for this study. Inclusion criteria were currently open clinical trials and trials focused on cancer in Africa. Studies that were suspended, terminated, completed, or withdrawn were excluded, because the primary objective of this study was to identify open oncology clinical trials in Africa.

### Data Retrieval and Management

A web-based review was used to identify public and private registries of oncology clinical trials based in Africa. The following data were collected from open oncology clinical trials in Africa: information source for the trials; country; type of cancer; a brief summary of study; sponsor(s) name and country; study type; estimated enrollment; allocation; official title of study; primary purpose of study; study start date and end date; and age and sex eligibility.

Two research coordinators affiliated with the Prostate Cancer Transatlantic Consortium and the 15 undergraduate trainees participating in the University of Florida Research Training Opportunities for Outstanding Leaders program as part of their service learning experience captured data. Each of the 17 data collectors was preassigned three to five countries to focus on for the data retrieval. Qualtrics (Provo, UT) survey software was used for data capture and management. Data validity and quality check were confirmed by a research coordinator at University of Florida. Data validation and quality check involved using the information source identified by data collectors to confirm the clinical trials in the database. In addition, the research coordinator checked for double entry to ensure unique entry.

### Data Analysis

The Qualtrics survey software was used for data analyses and reporting. Descriptive analyses, including frequency analysis and descriptions of findings, were used to summarize findings.

## RESULTS

**FIG 1:**
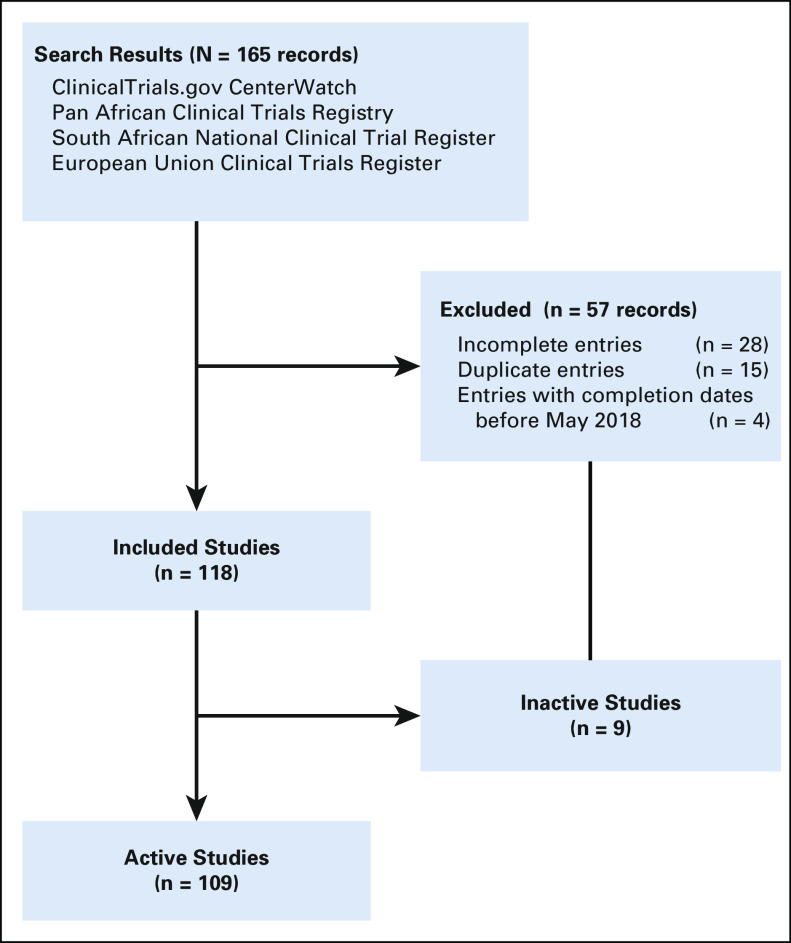
Flowchart of clinical trials selection.

Figure 1 is a summary of the clinical trials search for Africa. A total of 118 open clinical trials were identified. Of the 118 studies included, all but nine were validated as currently active. Active studies (n = 109), included studies that were registered and approved, and either were not yet recruiting, currently recruiting, or no longer recruiting but still ongoing (Table 1). If the study had not reached its primary completion date and had not been updated within the last 2 years, it was classified as of unknown status.

**TABLE 1:**
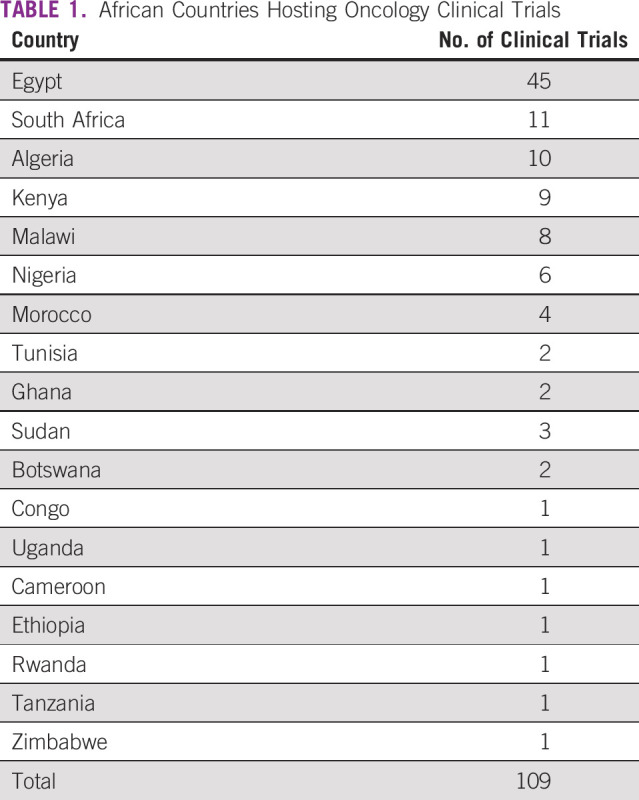
African Countries Hosting Oncology Clinical Trials

**FIG 2:**
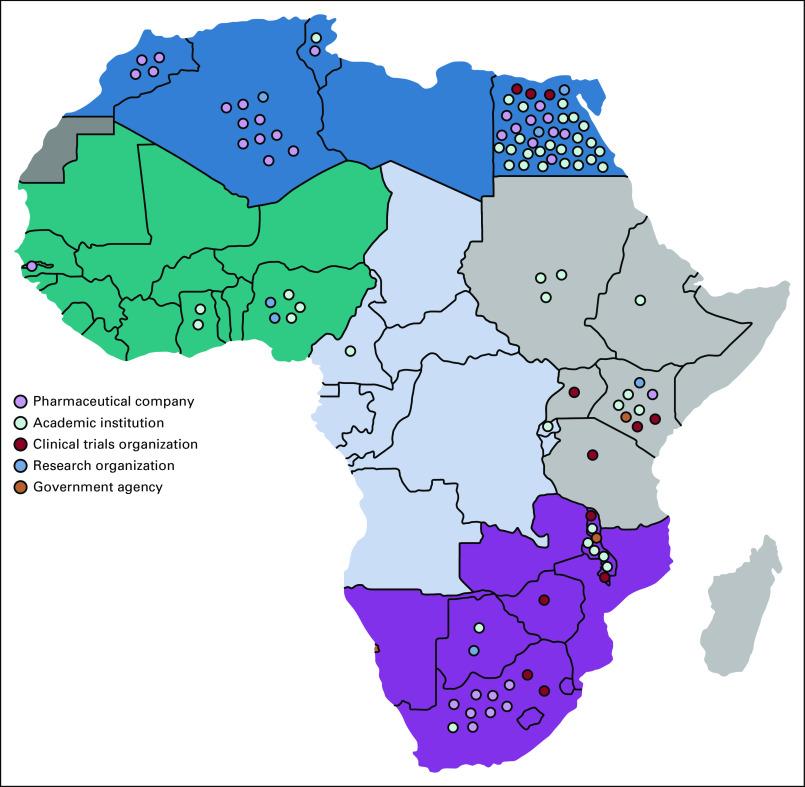
Landscape of oncology clinical trials in Africa.

Of the 54 countries in Africa, 20 hosted oncology clinical trials (Fig 2). The leading country was Egypt (n = 45 trials), followed by South Africa (n = 11), Algeria (n = 10), and Kenya (n = 10). North Africa (Egypt, Algeria, Morocco, Tunisia, and Sudan) was the densest region, with 63 clinical trials. Central Africa (Cameroon and Congo) had the least representation of all African regions (n = 3 clinical trials).

Table 2 summarizes the numbers of clinical trials by cancer type. The most common type of cancer for clinical trials in Africa is breast cancer (n = 41), followed by cervical (n = 13), and lung cancer (n = 10). Twelve studies were listed as “any” or “other,” denoting trials that included patients diagnosed with any type of cancer and designed to investigate oxidative stress in surgery, symptom control due to disease, symptom control as a result of chemotherapy, delay in diagnosis and treatment, febrile neutropenia complications after chemotherapy, pain control, physical activity and healthy diet, and physical rehabilitation in patients with cancer.

**TABLE 2:**
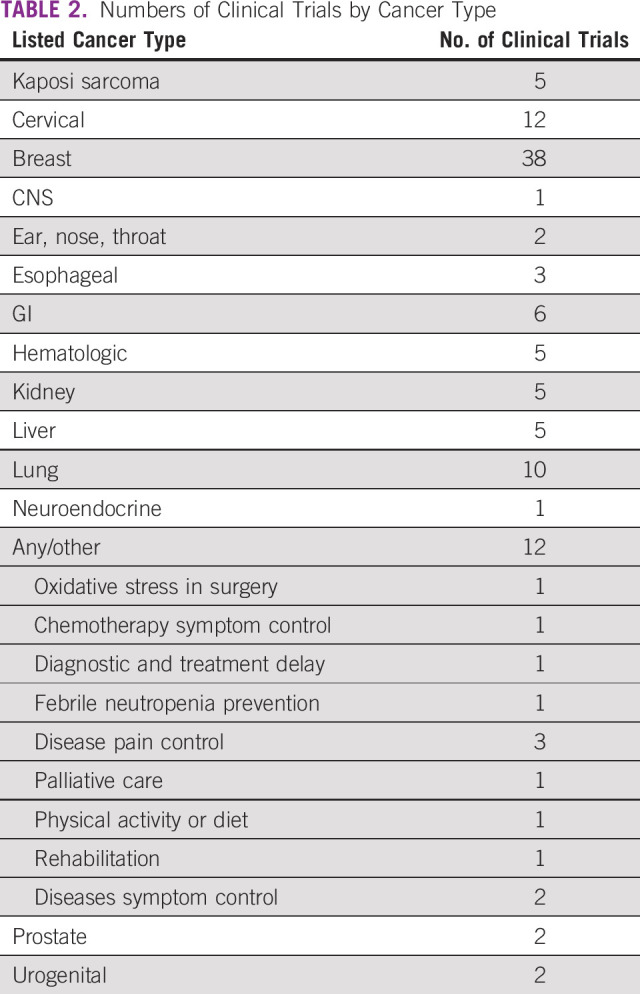
Numbers of Clinical Trials by Cancer Type

**FIG 3:**
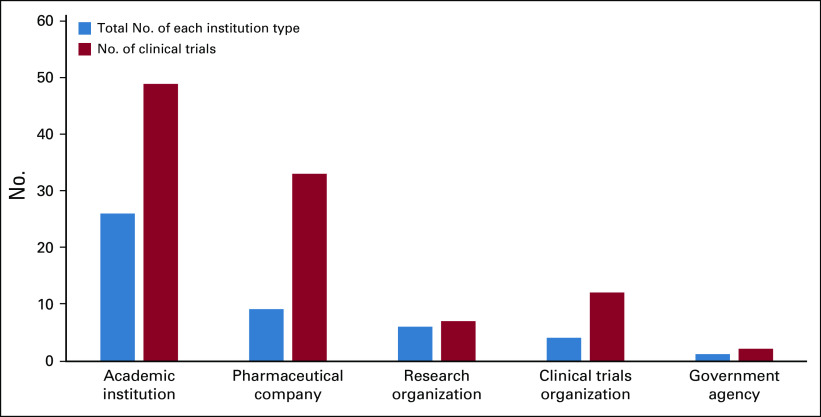
Number of clinical trials versus institution type. Does not include the six clinical trials listed with individual investigators as sponsors.

Table 3 and Figure 3 summarize the number of clinical trials by sponsor and country. Of the 52 institutions hosting oncology clinical trials in Africa, Hoffmann-La Roche, a Swiss pharmaceutical company, sponsored the most clinical trials (n = 19). Overall, the pharmaceutical industry was represented by a total of nine companies and conducted the second highest number of clinical trials (n = 34; Table 4). Academic institutions, defined as sole universities and university-affiliated hospitals, were the most represented type of sponsor (n = 28 academic institutions), and sponsored the most clinical trials in Africa (n = 52). Eight research organizations sponsored a total of 10 clinical trials.

**TABLE 3:**
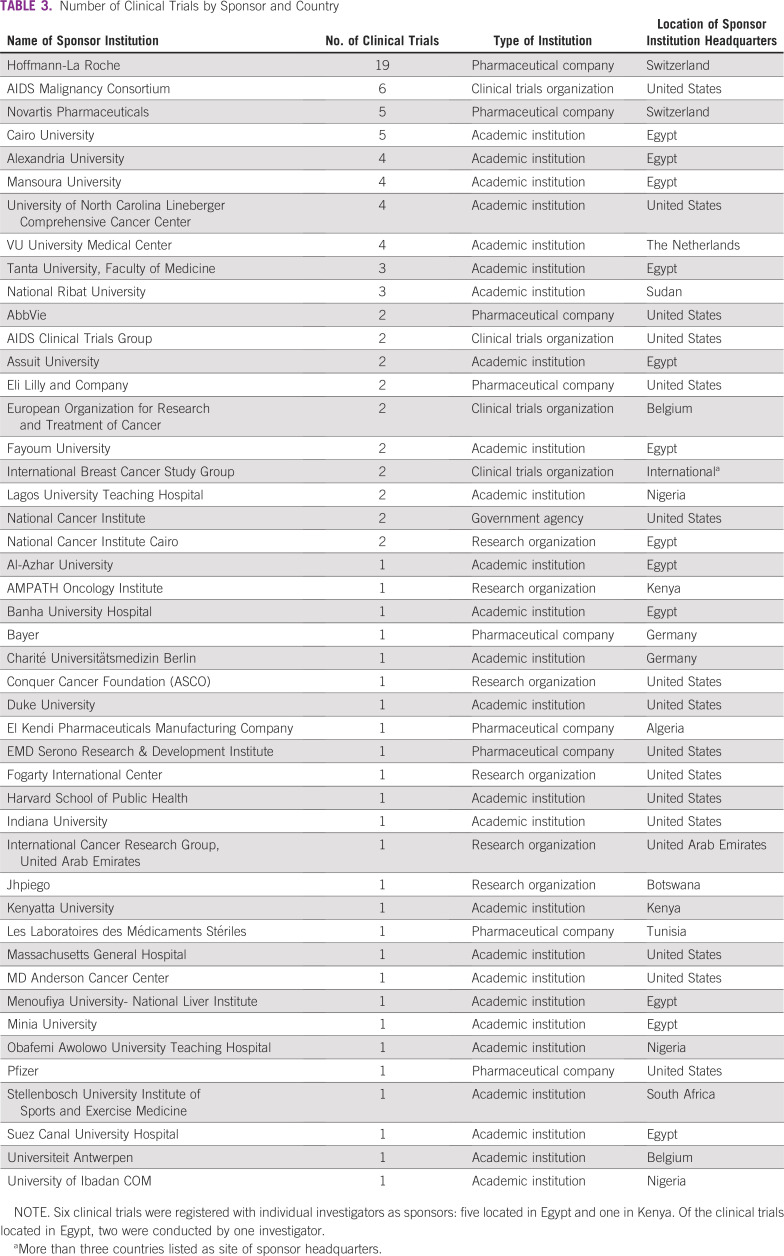
Number of Clinical Trials by Sponsor and Country

**TABLE 4:**
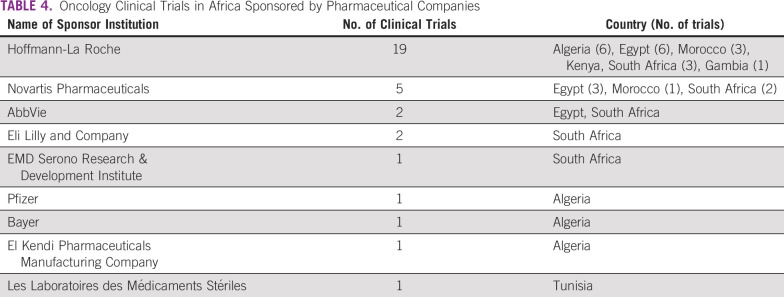
Oncology Clinical Trials in Africa Sponsored by Pharmaceutical Companies

**FIG 4:**
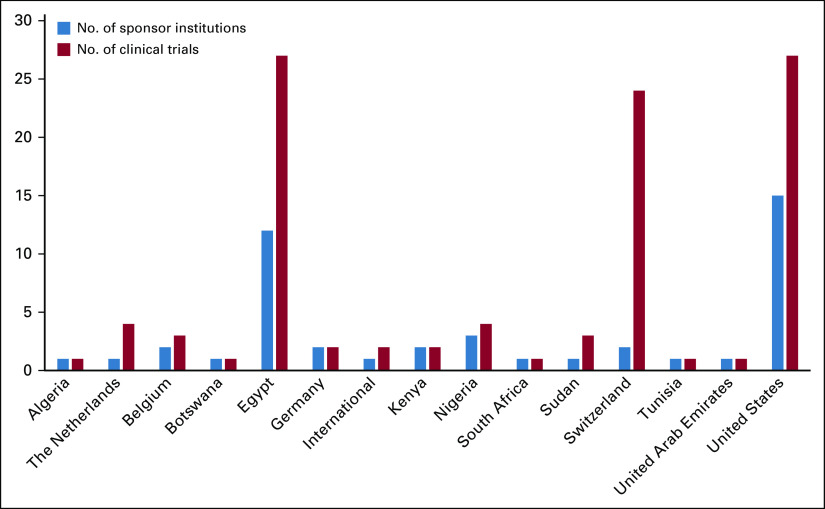
Number of sponsor institutions and clinical trials per country. Total does not include the six clinical trials listed with individual investigators as sponsors.

Overall, 15 institutions that sponsored oncology clinical trials in Africa were headquartered in the United States (Fig 4). Egypt followed with 12 institutions. Institutions based in the United States were responsible for 27 clinical trials; the same number was reported for all institutions based in Egypt. Both countries were responsible for the implementation of 54 of 118 cancer clinical trials performed in Africa. Twenty-five clinical trials were supported by two institutions in Switzerland, 19 of which were funded by Hoffmann-La Roche.

## DISCUSSION

In general, this study revealed a dearth of oncology trials in most of Africa despite the recognized high burden of cancer on the continent. Thus, there is underrepresentation of Africans in oncology clinical trials, which is similar to the underrepresentation of populations of African ancestry in high-income countries such as the United States.^17^ Unfortunately, the underrepresentation of Africans in oncology clinical trials results in disparity in access to high-quality health care and reduces the generalizability of research findings, especially because postmarketing clinical trials are few in Africa.

This study was conducted to answer three questions. The first research questions was, what are the public and private registries for oncology clinical trials in Africa? We found that the most effective registries for oncology clinical trials in Africa were ClinicalTrials.gov, CenterWatch, Pan African Clinical Trials Registry, South African National Clinical Trial Register, and European Union Clinical Trials Register. It took 18 people with highly reliable Internet access to identify and validate active trials through these sites. It is unrealistic to expect that oncology providers and patients in Africa will be able to easily access these trials. Thus, there is a need to build an African Virtual Platform for Oncology Clinical Trials, which will provide easy access for providers and patients. Funded by the Carnegie African Diaspora Fellowship award, this study was part of a larger project to establish the African Virtual Platform for Oncology Clinical Trials.

The second research question for this study was, who are the sponsors of oncology clinical trials in Africa? The top sponsor of oncology clinical trials in Africa was academic institutions, especially institutions in the United States. It is highly commendable for these institutions to invest in cancer research in Africa. Egypt leads this growth with 27 investigator-led trials out of a total of 45 trials. The remaining 18 trials in Egypt had pharmaceutical sponsors, indicating either a level of confidence in the ability to run trials to GCP standards or fewer obstacles to the operational processes for conducting trials. On the other hand, South Africa had only one investigator-led trial and 10 pharmaceutical trials. All the eight trials in Malawi were sponsored by pharmaceutical companies.

It is disappointing to note that pharmaceutical companies, which have the most to gain from oncology therapeutic interventions in Africa, are not investing in the health of African populations. We found an interesting pattern of regional clusters of trials supported by pharmaceutical companies by countries. The majority of these studies were located in six countries. This begs the question of what is providing the confidence for the predominant sponsors, such as Hoffman-La Roche and Novartis, to be present in these countries. Countries like Egypt, Algeria, Kenya, and South Africa, which have the highest numbers of clinical trials, have presumably invested more heavily in their overall research capacity and infrastructure development to host and manage clinical trials. In principle, collaboration with countries with an established track record would help improve the ability of emerging countries to host clinical trials.

In support of our findings, a report from Wemos Health Unlimited^18^ revealed that Egypt is second only to South Africa on the African continent in terms of the number of international pharmaceutical-company sponsored clinical trials it hosts. Over 3 years (2008 to 2011), the number of trials in Egypt nearly tripled, with more than half of all trials in Egypt being cancer trials by 2016. Reasons given by the authors included: (1) fast-growing, largely uninsured and treatment-naïve population; (2) large pool of diseases within population; (3) attractive research infrastructure; (4) lower costs for conducting trials; and (5) no official national law on clinical trials. The authors raised ethical concerns particularly for cancer trials, noting that patient acquiescence to enrollment in the trials may be due to the exorbitantly high price of cancer drugs. They estimated cancer care cost to be approximately EGP 50,000 (US$5,600) per month. With this high cost, participating in clinical trials provides free care for patients.^18^

Sponsor confidence resides in the presence of established IRBs, Medicines Regulatory Authorities (MRAs), Clinical Trial Units, and established GCP quality management systems. All these priorities require infrastructure and human capacity. Exploration of the IRB and MRA support for the top six countries with the highest number of cancer trials in this study revealed that all are supported by identified IRBs. For example, Egypt has 23 registered IRBs, Algeria has 50, South Africa has 30, and Nigeria has 26. Support for the development of national IRBs and MRAs has been forthcoming from organizations such as the European & Developing Countries Clinical Trials Partnership (EDCTP), which has rolled out an extensive program of funding for these projects. A recent EDCTP/Pan African Clinical Trial Alliance workshop revealed much has been achieved in those countries supported by grants, but there remain countries with gaps in this critical area of ethical and regulatory oversight.

International funding for oncology clinical trials in Africa is predominantly from EDCTP, the Bill & Melinda Gates Foundation, and the US National Institutes of Health (NIH). The NIH funding of trials is strongly focused in South Africa. This needs to shift, not only in disease focus (currently infectious diseases) but also to a more representative sample of African countries, especially sub-Saharan African countries. This will require generating funder confidence in trial site capacity to carry out trials to GCP standards and to manage funding with due diligence. A logical direction for building confidence in oncology trials lies in leveraging established units running trials in infectious diseases. It is critical that sponsors of trials in Africa move toward a partnership with departments of health and academic institutions as a means of widening the current research landscape to include trials in oncology.

Twelve trials were conducted by four clinical trial organizations, institutions with distinct missions to study disease biology and therapies solely through the context of clinical trials. Two of these organizations are dedicated to cancer; the remaining two focus on HIV and its complications, including AIDS-related cancer. Seven clinical trials were sponsored by individual investigators or health care professionals. In addition, one local hospital (Tenwek Hospital in Kenya) funded one clinical trial and the NIH sponsored two clinical trials.

The third research question we set out to answer in this study was, what are the common cancer types for oncology clinical trials in Africa? The top four cancer types for oncology clinical trials in Africa are breast, cervical, lung, and GI cancers. Kaposi sarcoma, hematologic, kidney, and liver cancers tied at number 5. According to the WHO 2018 GLOBOCAN data, the top five cancer types in Africa based on new cases are breast, cervix, prostate, liver, and colorectal cancers. Breast (n = 38) and cervical (n = 12) cancers are relatively well represented in oncology clinical trials in Africa. In comparison, there are two prostate cancer clinical trials, five liver cancer clinical trials, and no colorectal cancer clinical trial. The burden of cancer in African countries should be considered in developing priorities for oncology clinical trials in Africa.

In conclusion, this study was focused on describing the current landscape of oncology clinical trials in Africa. The findings are limited to information available on the databases we searched for this study and based on the search terms we used. In addition, only information retrievable in English was included. Despite these limitations, this study provides critical findings on the current status of oncology trials in Africa and opportunities for improvement. Unfortunately, most cancer therapies are introduced to Africa without any clinical trials to confirm the treatment is safe or efficacious in the diverse African populations. This is the primary reason that some cancer therapies fail in Africans. The main concern of the regulatory bodies are post-trial access to treatment, accessibility of drugs often tested outside Africa to the general population after approval has been obtained, and cost of the drugs to the population.^18-23^ The heads of MRAs in Africa need to fully engage pharmaceutical companies and revisit the policies on the introduction of new oncology therapies to African countries. In addition, clinical trials are needed for currently available therapies to ensure they are safe and effective in the African population. In the era of personalized and precision health, it no longer suffices to assume that drugs developed in North America, Europe, or Asia will be effective in the African population.
